# Thieno[2,3-*b*]pyridine compounds potently inhibit prostate cancer growth and motility

**DOI:** 10.1530/EO-24-0082

**Published:** 2025-07-08

**Authors:** M A Alkheilewi, D A Leach, A Mohr, R M Zwacka, P Laissue, M Metodiev, C L Bevan, M Van Rensburg, L I Pilkington, D Barker, J Reynisson, G N Brooke

**Affiliations:** ^1^ School of Life Sciences, University of Essex, Colchester, UK; ^2^ Department of Surgery and Cancer, Imperial College London, London, UK; ^3^ School of Chemical Sciences, University of Auckland, Auckland, New Zealand; ^4^ Te Punaha Matatini, Auckland, New Zealand; ^5^ MacDiarmid Institute for Advanced Materials and Nanotechnology, Wellington, New Zealand; ^6^ School of Allied Health Professions and Pharmacy, Keele University, Keele, UK

**Keywords:** prostate cancer, therapeutics, multinucleation, therapy resistance

## Abstract

**Objective:**

Prostate cancer growth is dependent upon androgens and hence therapies often target this signalling axis. These therapies, for example the antiandrogen enzalutamide, are successful in the majority of men; however, resistance is inevitable and the tumour progresses to the castrate-resistant stage, a disease of unmet clinical need. Consequently, there is a great need for novel therapeutics for castrate-resistant prostate cancer. Thieno[2,3-*b*]pyridine compounds have shown promise as novel anti-cancer molecules, but little is known about their efficacy in prostate cancer. To address this, a panel of thieno[2,3-*b*]pyridine compounds was screened to identify those with cytostatic/cytotoxic activity in prostate cancer.

**Methods:**

The effect of the compounds upon prostate cancer proliferation and motility was assessed in a panel of cell lines representing different stages of the disease and non-tumorigenic controls. The effect of the compounds upon cell morphology and cell death was assessed using imaging and flow cytometry, respectively. The efficacy of the lead compound was also assessed in a patient-derived explant model.

**Results:**

The compounds were found to inhibit prostate cancer proliferation and motility, promote G2/M arrest, multinucleation and apoptosis. Importantly, treatment of patient-derived explants with the lead compound DJ160 demonstrated that the molecule inhibits prostate cancer proliferation, even in samples that appear to be resistant to enzalutamide.

**Conclusions:**

Thieno[2,3-*b*]pyridines therefore represent a potential therapy for prostate cancer, even when current therapies have failed.

## Introduction

Prostate cancer is the most commonly diagnosed cancer in men in the UK and it is a leading cause of cancer-related death (Smith-Palmer *et al.* 2019). Tumour growth is usually dependent upon the androgen receptor (AR) pathway and hence therapies for non-organ-confined disease target this signalling axis. This is achieved through inhibition of the AR directly, using antiandrogens (e.g. enzalutamide), or via the inhibition of androgen production (e.g. LHRH analogues). These therapies are successful in the majority of patients but invariably fail and the tumours progress to the aggressive castrate-resistant stage (Brooke & Bevan 2009). There are few therapeutic options for this stage of the disease and hence new methods to treat castrate-resistant disease are greatly needed.

Thieno[2,3-*b*]pyridines were previously identified through *in silico* screening as potential inhibitors of phosphoinositide-specific phospholipase C gamma (PLCγ) (Reynisson *et al.* 2009). However, it has now been established that the thieno[2,3-*b*]pyridines modulate several biological targets related to tumourigenesis: i) phospholipase C delta 1/3 (PLCδ1/3), deduced by the same cellular behaviour of the MDA-MB-231 breast cancer cell line upon administration of thieno[2,3-*b*]pyridines and following knock-down of the PLCδ1/3 genes (Reynisson *et al.* 2016); ii) copper-trafficking antioxidant 1 (ATOX1) protein, the inhibition of which reduces the proliferation of cancer cells (Wang *et al.* 2015); iii) tyrosyl DNA phosphodiesterase 1 (TDP 1), a phospholipase D enzyme involved in repairing DNA damage (Arabshahi *et al.* 2015); iv) the colchicine binding site in tubulin (Romagnoli *et al.* 2013, Eurtivong *et al.* 2017), an established target for anticancer drugs; and v) adenosine A2A receptor (A_2A_AR) (Katritch *et al.* 2010), a G protein-coupled receptor (GPCR). It can therefore be stated that the thieno[2,3-*b*]pyridines are multi-targeting compounds and function through polypharmacology (Proschak *et al.* 2019). Despite these previous studies, relatively little is known about the effectiveness of thieno[2,3-*b*]pyridines in prostate cancer.

**Figure 1:**
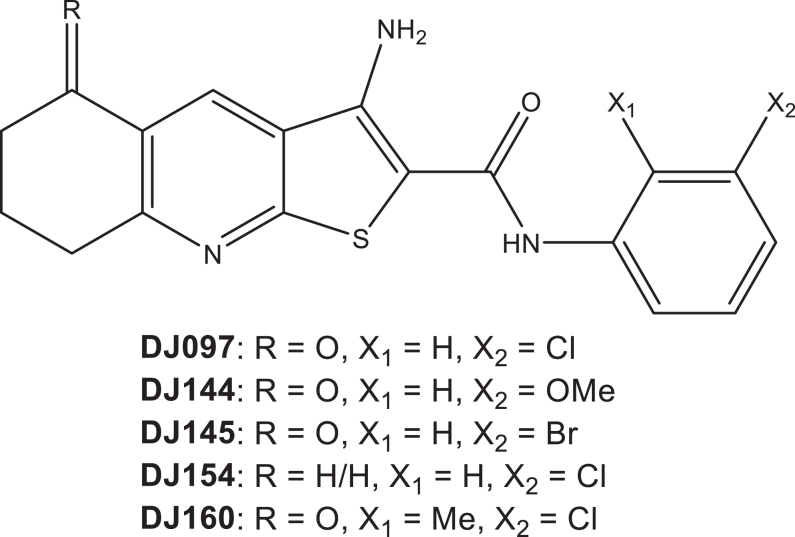
The molecular structures of the anticancer thieno[2,3-*b*]pyridines (3-amino-5-oxo-N-phenyl-5,6,7,8 tetrahydrothieno[2,3-*b*]-quinoline-2-carboxamide) selected for testing against prostate cancer.

We hypothesised that thieno[2,3-*b*]pyridines could be an effective treatment strategy for prostate cancer. The study therefore aimed to investigate the efficacy of five thieno[2,3-*b*]pyridines derivatives (molecular structures provided in Fig. 1) as novel therapeutic options for prostate cancer. Some of the compounds were found to have selectivity for prostate cancer cells and to be effective in a patient-derived explant model. These inhibitors therefore have the potential to be novel therapeutics for prostate cancer, including castrate-resistant disease.

## Methods

### Thieno[2,3-*b*]pyridine derivatives

Thieno[2,3-*b*]pyridines (DJ97, DJ144, DJ145, DJ154 and DJ160) were synthesised using previously reported methods (Hung *et al.* 2014, Leung *et al.* 2016, van Rensburg *et al.* 2017). Following synthesis, all compounds were confirmed to be ≥95% pure, as determined by NMR. For all experiments, the final DMSO concentration was 0.1%.

### Cell culture

Cell lines were obtained from the American Type Culture Collection (ATCC, USA). Lines were STR-authenticated by the ATCC and were frozen at a low passage. Cells were grown for a maximum of 6 months before a fresh aliquot was defrosted. All lines were cultured in RPMI medium supplemented with 10% FCS (HyClone Laboratories, USA) and penicillin-streptomycin-glutamine (PSG) (Brooke *et al.* 2011).

### Crystal violet proliferation assay

Cells were seeded at a density of 2 × 10^4^ in 96-well plates and left to adhere overnight before treatment with the inhibitors. After 72 h of treatment, cells were fixed in 2% paraformaldehyde for 1 h, washed 3× with ddH_2_O and left to dry. 0.04% crystal violet (dissolved in ddH_2_O) was added to each well and the plates incubated for 1 h. The plates were again washed 3× with ddH_2_O and left to dry. Crystal violet was solubilised with 10% acetic acid (1 h at room temperature with rocking) and the optical density of each well measured at 570 nm with a plate reader (CLARIOstar, BMG Labtech, Germany).

### Immunoblotting

Cells were lysed in RIPA buffer and lysates separated using SDS-PAGE as previously described (Leach *et al.* 2021). Membranes were probed with primary antibodies specific for PLCγ1 and PLCγ2 (D9H10 and E5U4T, Cell Signalling Technology, USA), the AR (ER179(2), Abcam, UK) and α-tubulin (B-5-1-2, Sigma Aldrich, USA). Immobilon Forte HRP substrate (Merck Millipore, USA) and an Odyssey XF Imaging System (LiCOR, USA) were used for visualisation.

### PLC activity assay

PC3 cells were grown in 10 cm dishes and treated with different concentrations of DJ160 for 24 h. Cells were collected and enzymatic activity quantified using a PLC Activity Assay Kit (Sigma Aldrich), following the manufacturer’s instructions.

### Cell cycle analysis

Cells were seeded in 12-well plates, incubated overnight and treated with the inhibitors. The cells were incubated for 72 h before harvesting. Cells were pelleted (200 ***g***, 5 min) and washed with PBS. The wash was removed and cells fixed with 70% EtOH, added dropwise with agitation. The cells were incubated at 4°C overnight, pelleted, washed with PBS and resuspended in PBS containing PI and RNase A, as previously described (Brooke *et al.* 2011). The percentage of cells in each phase of the cell cycle was subsequently quantified using an Accuri C6 Flow Cytometer (BD Biosciences, USA).

### DNA hypodiploidy staining

Cells were seeded in 12-well plates and left to adhere overnight before treatment for 24, 48 or 72 h. For Z-VAD-FMK (Santa Cruz, USA) experiments, the pan-caspase inhibitor was added (20 μM) 25 min before the addition of the thieno[2,3-*b*]pyridine compounds. Spent media, washes and trypsinised cells were collected into the same tube and cells pelleted using centrifugation (1,100 ***g***, 3 min). Cells were washed with PBS and Nicoletti buffer added, as previously described (Mohr *et al.* 2019). Cells were immediately vortexed (medium speed for 10 s) and incubated at 4°C for 1 h in the darkness. The percentage of cells undergoing apoptosis was subsequently quantified using an Accuri C6 Flow Cytometer.

### Propidium iodide exclusion assay

Cells were seeded in 12-well plates and left overnight to adhere. Cells were treated for 24, 48 or 72 h before being harvested. Spent media, washes and trypsinised cells were collected into the same tube. Propidium iodide was added to a final concentration of 10 μg/mL, the tube was vortexed and the sample read immediately using a flow cytometer.

### Caspase 3/7 assay

Cells were seeded in 96-well plates, treated and left for 72 h. Changes in caspase 3/7 activity were measured using a Caspase Glo kit (Promega, USA), following the manufacturer’s instructions. Caspase 3/7 activity was normalised to cell proliferation and quantified on a simultaneous plate using the crystal violet assay.

### Fluorescent imaging

Cells were seeded on glass coverslips and incubated overnight before treatment with the compounds. Cells were incubated for 24 h with the thieno[2,3-*b*]pyridines and fixed with 2% paraformaldehyde for 15 min at RT. Cells were washed 3 times with PBS and 0.1% Triton X-100 in PBS added for 10 min at RT. The washes were repeated and cells blocked with 1% BSA in 0.5% Tween for 30 min. Primary antibody (α-alpha-tubulin, clone B-5-1-2, Sigma Aldrich, USA) was diluted in the block buffer, added to the cells and incubated for 1 h. The cells were washed 3 times with PBS containing 0.5% Tween and secondary antibody applied (anti-mouse Alexa Fluor 568, Abcam, UK). Cells were incubated for 1 h in the darkness, washed, mounted on glass slides using Vectashield containing DAPI (Vector Labs, USA) and sealed using Fixogum (Marabu, Germany). Cells were visualised using an Eclipse Ti Widefield Fluorescent Microscope (Nikon, Japan). To calculate the percentage of cells with multinucleation, ten random fields of view were imaged per slide using low magnification (10×) and the number of cells with more than 1 nucleus counted. Alterations in cell size were quantified using Fiji (Schindelin *et al.* 2012).

### Single cell tracking

PC3 cells stably transfected with GFP (termed PC3-GFP) were seeded in 96-well plates (1 × 10^3^ per well) and incubated overnight. Cells were treated for 48 h with non-growth inhibitory concentrations of the inhibitors: 10 nM of compounds 144 and 160, and 100 nM of 97, 145 and 154. Single cell tracking (images acquired every 15 min for 24 h) was performed using a climate-controlled stage and cell motility assessed using the Fiji plugin TrackMate (Tinevez *et al.* 2017).

### Tumour explants

Explant experiments were undertaken using published methodology (Centenera *et al.* 2018). Biopsy tissue from prostate cancer patients was immediately placed in ice-cold medium straight from surgery. In the lab, tissue was dissected and seeded onto a sponge (SSP101010-32, Surgispon, Anser Medical) in 24-well plates. The explants were treated with compound 160 (100 nM) or the antiandrogen enzalutamide (10 μM) and incubated at 37°C for 48 h.

### qPCR

Explants were snap frozen until RNA was extracted using a Monarch RNA extraction kit (NEB), following disruption of frozen tissue utilising mechanical disruption and enzymatic digestion with proteinase K. Then, 2 μg of RNA was reverse transcribed using Precision Nanoscript2 RT Kit (Primer Design, UK). Changes in gene expression were measured using SYBR Green Fast Master Mix (Life Technologies, USA) and QuantStudio 7 Flex Real-Time PCR System (Applied Biosystems, USA). *PCNA* expression data were normalised to *L19, GAPDH* and *ACTB* data and the 2^(–^ΔΔ^CT)^ method used to calculate changes in expression. qPCR primer sequences are provided in Supplemental Table 1 (see section on Supplementary materials given at the end of the article).

### Immunohistochemistry

The explants were formalin fixed and wax embedded. Immunohistochemistry was performed to investigate the expression of cleaved caspase 3 (Cell Signalling 9661S (1/200)) as a marker of apoptosis. Protein expression was detected using the REAL EnVision Detection System (Peroxidase/DAB, K500711-2, DAKO).

## Results

### Thieno[2,3-*b*]pyridines potently inhibit prostate cancer cell line proliferation but do not appear to act via PLC

**Figure 2:**
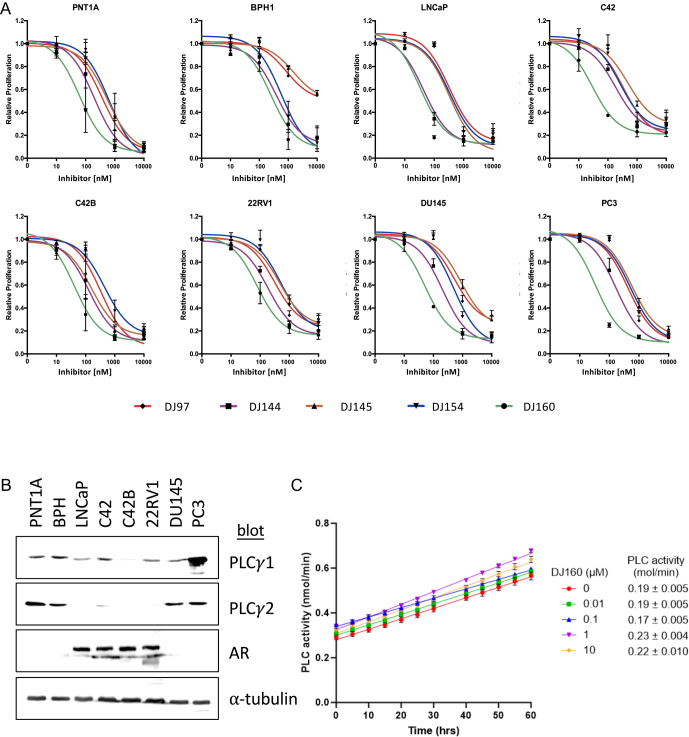
The thieno[2,3-*b*]pyridine compounds inhibit prostate cancer cell lines. (A) The non-tumorigenic controls PNT1A and BPH1, and prostate cancer cell lines LNCaP, C42, C42B, 22RV1, DU145 and PC3 were seeded 24 h before treatment with a dose range of the thieno[2,3-*b*]pyridine compounds (DJ97, DJ144, DJ145, DJ154 and DJ160). After 72 h of treatment, cells were fixed, stained with crystal violet and absorbance measured using a spectrophotometer microplate reader (FLUOstar Omega) at 490 nm. Mean ± SE of three independent repeats (six replicates for each concentration per experiment). (B) Western blotting was performed on lysates from the different cell lines and membranes probed with antibodies specific for PLC*γ*1, PLC*γ*2, AR and ⍺-tubulin. (C) PC3 cells were seeded in 10 cm plates and treated with a dose range of DJ160 for 24 h. Cells were collected and PLC activity measured, and enzymatic activity calculated. Mean of 3 repeats ± SD.

To explore the inhibitory activity of thieno[2,3-*b*]pyridines in prostate cancer, the anti-proliferative activity of five compounds was assessed across a range of cell lines representing different stages of the disease: non-tumorigenic (PNT1A and BPH1), hormone sensitive (LNCaP) and castrate resistance (C42, C42B, 22RV1, DU145, PC3). All of the inhibitors tested successfully blocked proliferation in a dose dependent manner (Fig. 2A), with compound DJ160 being the most potent across all lines. Importantly, the compounds show specificity for the cancer cell lines compared to the non-tumorigenic control line BPH1. For example, the IC_50_ values for compounds DJ97 and DJ145 were in the μM range for BPH1 and were in the nM range for the PCa cell lines, with the exception of DU145 (Table 1).

**Table 1:** The IC_50_ values (nM) of the thieno[2,3-*b*]pyridine compounds demonstrate that DJ160 is the most potent inhibitor in prostate cancer cell lines. IC_50_ values were calculated from the sigmoidal range response curves for the cell lines PNT1A, BPH, LNCaP, C42, C42B, 22RV1, DU145 and PC3 after treatment with the thieno[2,3-*b*]pyridine compounds (DJ97, DJ144, DJ145, DJ154 and DJ160). Data are shown as mean ± SD.

	Cell line		IC_50_ (nM)				
			DJ97	DJ144	DJ145	DJ154	DJ160
Non-tumorigenic	PNT1A	Mean	324.0	238.2	708.0	902.0	82.6
		SD	44.3	136.5	140.4	748.0	35.4
	BPH1	Mean	3,551.4	442.8	4,361.9	806.0	151.1
		SD	5,340.2	275.8	4,913.3	523.2	60.3
AR positive, hormone sensitive to insensitive	LNCaP	Mean	481.8	59.4	267.6	451.7	44.9
		SD	123.0	16.5	177.7	164.3	12.7
	C42	Mean	334.0	187.0	468.0	246.0	29.0
		SD	6.8	36.7	163.2	74.0	17.8
	C42B	Mean	333.0	150.0	230.3	512.0	54.0
		SD	24.6	136.5	182.2	305.0	44.0
	22RV1	Mean	305.5	184.7	499.0	520.7	100.0
		SD	54.4	39.6	72.8	125.6	49.3
AR negative	DU145	Mean	1,396.0	271.4	1,959.6	660.4	83.9
		SD	640.0	52.4	755.5	140.3	16.5
	PC3	Mean	572.8	214.1	672.8	723.3	46.5
		SD	128.9	88.6	41.3	99.8	7.3

As mentioned previously, the thieno[2,3-*b*]pyridines were identified through *in silico* screening as potential inhibitors of PLCγ (Reynisson *et al.* 2009). However, more recent research has suggested that they have polypharmacology and inhibit a variety of targets. To investigate this further, PLCγ1 and PLCγ2 expression was analysed across a panel of prostate cell lines representing different stages of the disease (Fig. 2B). PLCγ1 was found to be fairly uniformly expressed across the cell lines, with the exception of PC3, where it was found to be highly expressed. In contrast, PLCγ2 was found to have an inverse correlation with the AR, with levels lower in LNCaP and its derivatives (C42 and C42B) and 22RV1. Importantly, expression of the PLCγs does not correlate with drug sensitivity (Table 1). To see if the most potent compound, DJ160, is able to inhibit PLC activity, enzymatic assays were performed (Fig. 2C). DJ160 was found to have little effect upon pan-PLC activity, again suggesting that these compounds exert their effects via other targets.

### Thieno[2,3-*b*]pyridines promote G2/M cell cycle arrest and apoptosis

**Figure 3:**
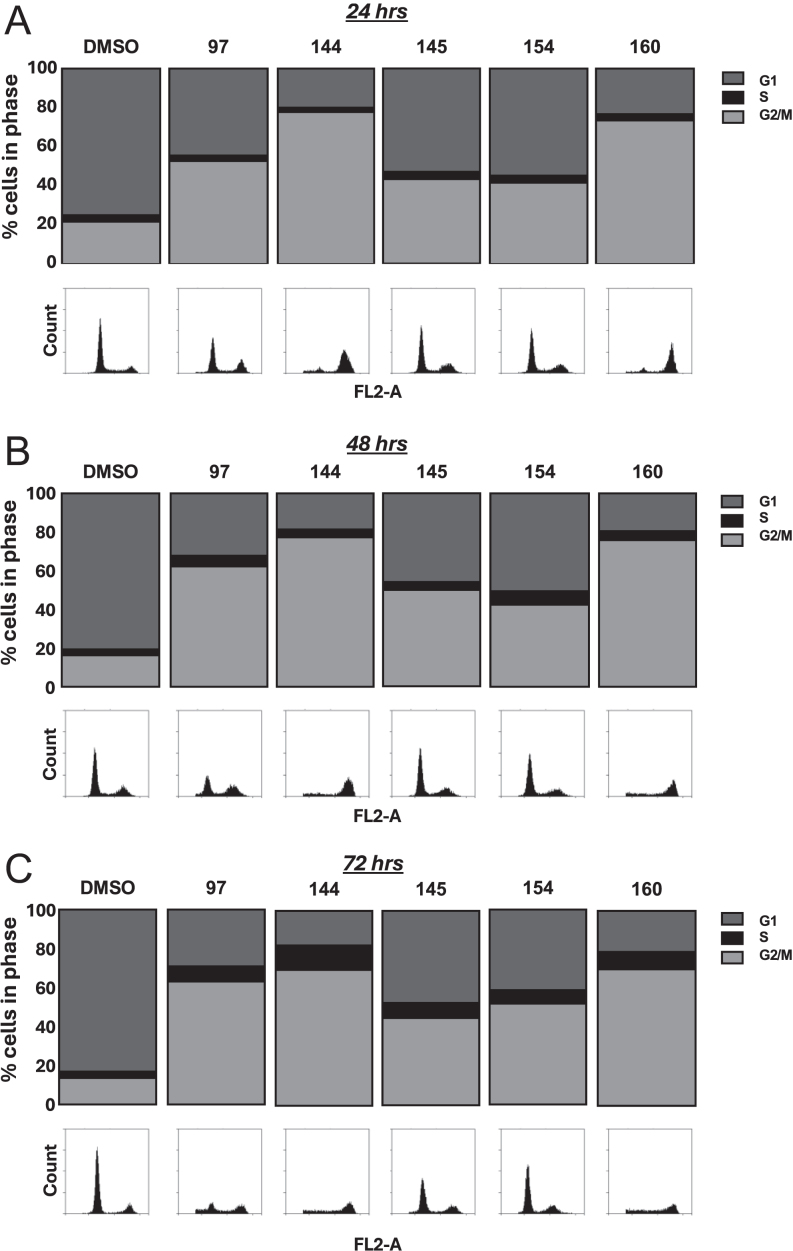
The thieno[2,3-*b*]pyridine compounds promote G2/M cell cycle arrest in PC3 cells. PC3 cells were seeded at 30% confluence for 48 h and treated with 1 μM of the compounds (97, 144, 145, 154 and 160) or DMSO for (A) 24, (B) 48 and (C) 72 h. Cells were PI stained and DNA content measured using flow cytometry. The percentage of cells in each phase was calculated and representative histograms generated using the ACCURI C6 software (BD Biosciences). Data are the mean of three independent repeats.

To investigate if the thieno[2,3-*b*]pyridines promote growth arrest, the aggressive metastatic PC3 cell line was treated with the inhibitors for 24, 48 and 72 h and flow cytometry analysis was performed to investigate cell cycle progression (Fig. 3A, B, C). As expected, in the absence of an inhibitor, the majority of the cells were in the G1 phase throughout the time course. After 24 h all of the inhibitors had started to induce G2/M arrest (Fig. 3A), with compounds DJ144 and DJ160 having the most pronounced effect. At the latter time point (72 h), there was a reduction/loss of cells present in the different phases of the cell cycle, suggesting that the majority had undergone cell death (Fig. 3C, see histograms).

**Figure 4:**
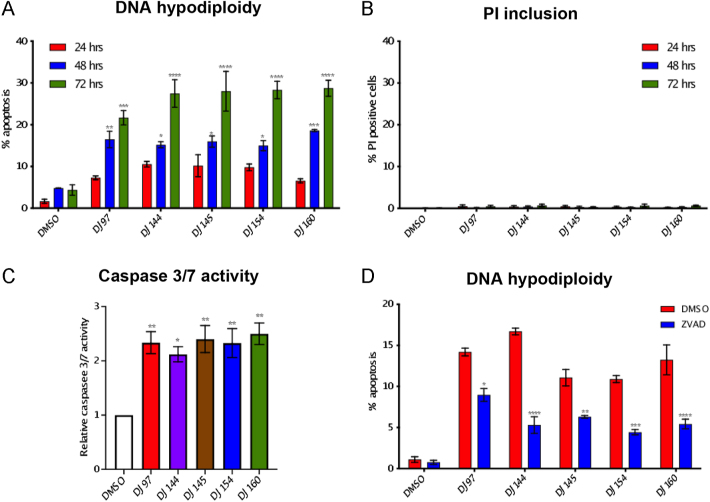
The thieno[2,3-*b*]pyridine compounds promote apoptosis in PC3 cells. PC3 cells were seeded at 30% confluence and incubated for 24 h before treatment with 1 μM of the compounds (DJ97, DJ144, DJ145, DJ154 and DJ160) or DMSO for different time points (24, 48 and 72 h). Cells were harvested and (A) DNA hypodiploidy (apoptosis) and (B) PI inclusion (caspase-independent cell death) measured using flow cytometry. (C) Caspase-Glo assays were performed to assess caspase 3/7 activity in PC3 cells treated with 1 μM of the compounds for 48 h. Luciferase activity was measured using a FLUOstar Omega microplate reader and made relative to control (DMSO) treated cells. (D) PC3 cells were seeded at 30% confluence and incubated for 24 h. Cells were treated with 20 μM Z-VAD-FMK for 25 min before the addition of the inhibitors for 48 h. DNA hypodiploidy assays were performed to quantify the number of cells undergoing apoptosis. Mean ± 1 SE of three independent experiments. ANOVA ****P < 0.00001, ***P < 0.0001, **P < 0.001 and *P < 0.01.

To see if the compounds also induce cell death, DNA hypodiploidy (used to measure apoptosis) and PI exclusion (quantification of necrosis/necroptosis) assays were performed (Fig. 4A and B). In the absence of the inhibitors, apoptosis remained at approximately 5% over the time course. All of the drugs significantly increased apoptosis in a time dependent manner (Fig. 4A). The PI inclusion assay demonstrated that the inhibitors do not induce necrosis/necroptosis (Fig. 4B). To confirm that cell death is via apoptosis (a caspase-dependent process), Caspase 3/7-Glo assays (Fig. 4C) and Z-VAD-FMK inhibitor experiments were performed (Fig. 4D). As expected, the thieno[2,3-*b*]pyridines increased caspase 3/7 activity more than 2-fold. Furthermore, pre-treatment (25 min) with the pan-caspase inhibitor Z-VAD-FMK before treatment with the compounds led to a significant reduction in the percentage of cells undergoing apoptosis. The inhibitors therefore block cell cycle progression through G2/M arrest and lead to apoptosis.

### Thieno[2,3-*b*]pyridines promote multinucleation and increase cell size

**Figure 5:**
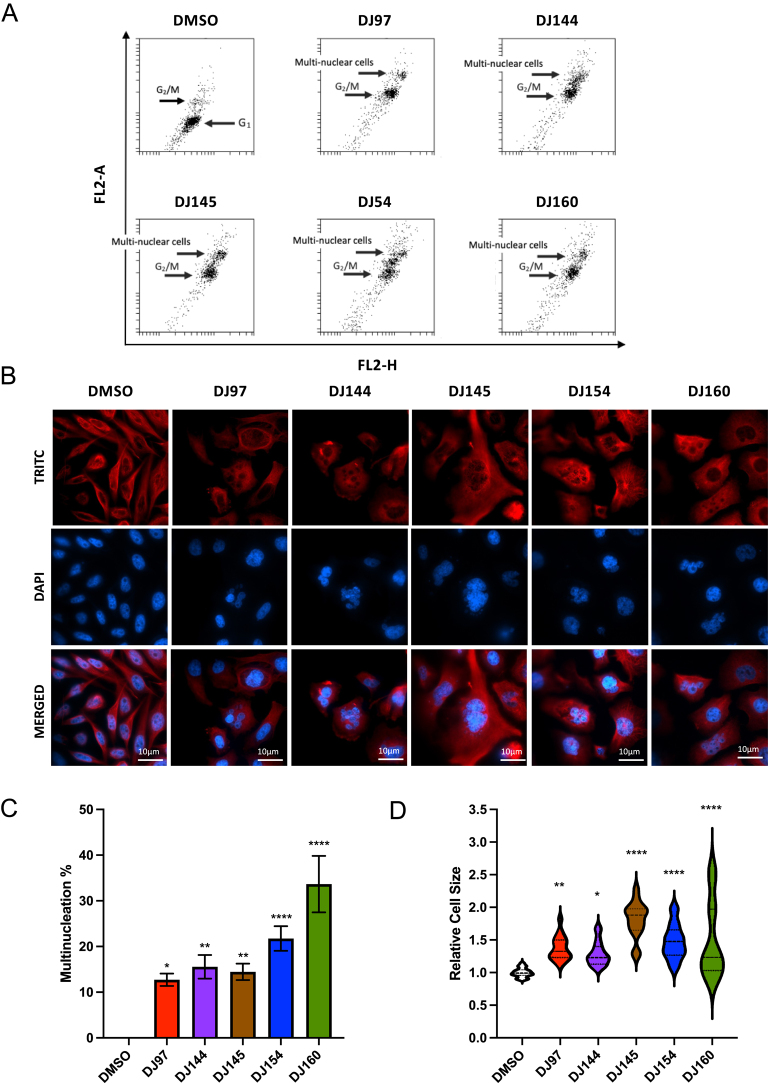
The thieno[2,3-*b*]pyridine compounds promote multinucleation and lead to an increase in cell size. (A) PC3 cells were seeded at 30% confluence for 48 h then treated with 1 μM of the compounds (DJ97, DJ144, DJ145, DJ154 and DJ160) or DMSO. Cells were PI stained and DNA content was measured using flow cytometry. The population of cells in G2/M and with potential multinucleation are highlighted. Dot blots were generated using Accuri C6 BD Biosciences software. (B) PC3 cells were seeded on coverslips and treated with 1 μM of the drugs (97, 144, 145, 154 and 160) or DMSO for 24 h. Cells were fixed with 4% paraformaldehyde and methanol, and immunofluorescence was performed using an antibody specific for α-tubulin and a secondary α-TRITC antibody. Nuclear staining = 4′,6-diamidino-2-phenylindole (DAPI) in blue. Confocal microscopy was performed and five random images were taken for each treatment to quantify (C) percentage multinucleation and (D) relative cell size. ANOVA ****P < 0.00001, **P < 0.001, *P < 0.01. Mean ± SE.

As described previously, in the absence of the inhibitors (DMSO-treated), the cells were predominantly in G1 and a smaller proportion were in G2/M. Following the addition of the thieno[2,3-*b*]pyridines, the majority of cells were found to be in G2/M. Interestingly, there was also a pool of cells with high FL2-Area and FL2-Height (Fig. 5A), which could be an indication that the inhibitors promote multinucleation. To investigate this, immunofluorescence was performed using an antibody specific for α-tubulin and nuclei were stained with 4′,6-diamidino-2-phenylindole (DAPI). In the absence of the thieno[2,3-*b*]pyridines, all cells were found to be mononuclear (Fig. 5B). Treatment with the compounds appeared to result in an enlargement in cell size and a number of cells were found to be multinuclear. To quantify this, ten random fields of view were visualised under low magnification and the percentage of cells with multinucleation and the relative size of the cells were calculated (Fig. 5C and D). In the absence of inhibitors, no cells were found to have multinucleation, whereas more than 10% of cells were found to be multinucleated in response to the drugs. Compound DJ160 had the most significant effect, increasing the number of cells with multinucleation to more than 30%. Cell size was also found to be significantly increased by all of the compounds (Fig. 5D).

### Thieno[2,3-*b*]pyridines inhibit cell motility

**Figure 6:**
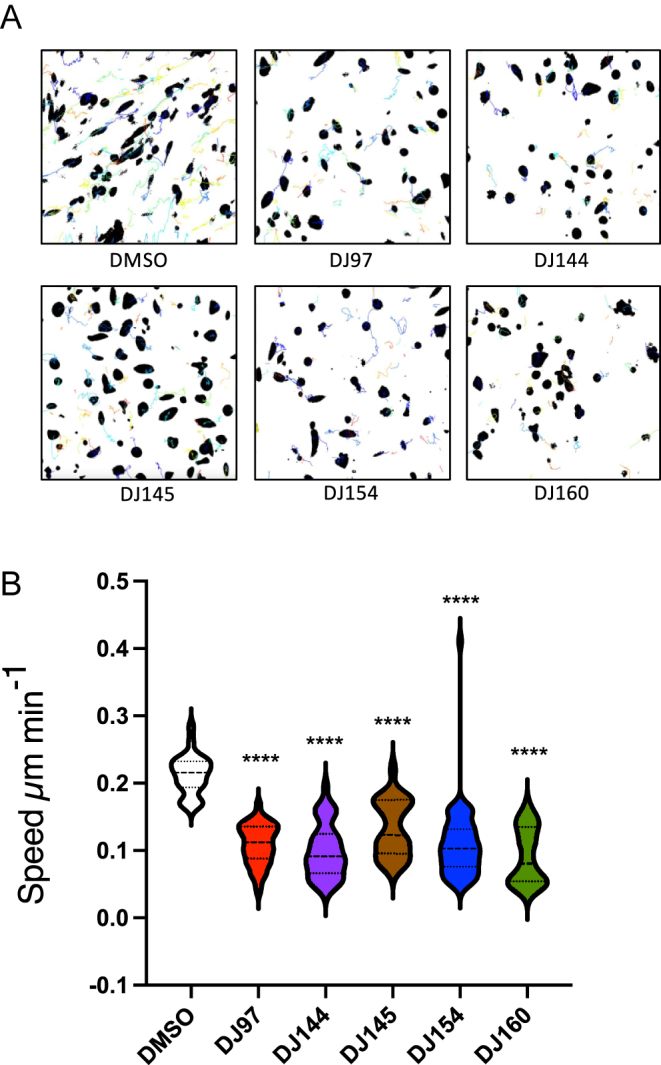
The thieno[2,3-*b*]pyridine compounds reduce cell motility. (A) PC3-GFP cells were incubated for 24 h before treatment with 10 nM of compounds 144 and 160 and 100 nM of 97, 145 and 154 or DMSO for 48 h. Images for all treatment and control were acquired every 15 min for 24 h. Mean of cell speed (μm/frame) was calculated from time-lapse sequences. (B) Images show tracks of PC3-GFP cells after treatment with the thieno[2,3-*b*]pyridine inhibitors. Images were acquired using TrackMate (ImageJ). ANOVA ****P < 0.00001. Mean ± SE.

Approximately 90% of cancer deaths can be attributed to metastases and therefore molecules that block cancer cell motility are likely to have significant impact upon disease progression. To see if the inhibitors affect the motility of prostate cancer cells, single cell tracking using time-lapse photography was performed on PC3 cells stably transfected with GFP. Cells were treated for 48 h with non-toxic concentrations of the compounds (calculated from the proliferation assays, Fig. 2): 10 nM for DJ144 and DJ160; 100 nM for DJ97, DJ145 and DJ154. Using fluorescent microscopy, images were taken every 15 min for 24 h and the effect upon cell motility visualised using TrackMate (ImageJ). In the absence of the treatments, the cells were found to migrate throughout the field of view and this movement appeared to be reduced in the presence of the inhibitors (Fig. 6A). Cell migration was subsequently quantified (Fig. 6B) and similar to previous reports (Lazarini *et al.* 2013), PC3 cell migration in the absence of an inhibitor was found to be approximately 0.2 μm min^−1^. The inhibitors significantly reduced cell motility to approximately 0.1 μm min^−1^. In addition to inhibition of proliferation and induction of apoptosis, the compounds therefore also block cell motility at sub-lethal concentrations.

### Thieno[2,3-*b*]pyridines have anti-proliferative effects in patient-derived explants

We have demonstrated that the thieno[2,3-*b*]pyridines block prostate cancer proliferation and motility. Furthermore, these molecules promote multinucleation, G2/M arrest and apoptosis in PC3 cells. To assess the inhibitors in a more physiologically relevant model, the efficacy of the compounds was assessed in patient-derived explants. Tumour tissue from four patients was dissected into 1 mm^3^ sections and cultured in duplicate on a pre-soaked gelatin sponge. Samples were treated with 10 μM enzalutamide or 100 nM DJ160. DJ160 was chosen for these experiments due to its potency in the previous experiments. The samples were treated with the drugs for 48 h and then divided into two portions. One portion was used for qPCR analysis while the other portion was formalin-fixed for immunohistochemistry.

**Figure 7:**
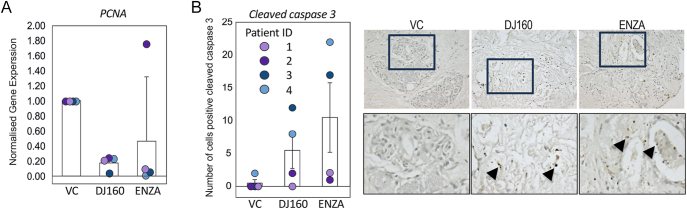
Thieno[2,3-*b*]pyridine DJ160 inhibits activity in cultured human prostate tumours. Tumours were dissected into 1 mm^3^ pieces then treated with the drugs (100 nM DJ160 and 10 μM enzalutamide (ENZA)) or vehicle control (DMSO, VC) and incubated for 48 h. (A) RNA was extracted and qPCR was performed to quantify the expression of *PCNA*, used as a marker of proliferation. (B) Samples were formalin-fixed and immunohistochemistry performed for cleaved caspase 3 and the number of cells per field counted. Example images for patient 3 are provided (20× magnification).

To investigate if DJ160 inhibits the growth of patient tumours, qPCR was performed for *PCNA* and used as a marker of proliferation (Dai *et al.* 2014). The compound reduced PCNA expression by approximately 80% compared to untreated samples and was effective in all patient explants (Fig. 7A). Enzalutamide had similar efficacy for three out of four tumours; however, sample 2 appears to be resistant to this antiandrogen. To see if DJ160 also promotes apoptosis in these samples, the effect of the drugs on caspase 3 cleavage was assessed using immunohistochemistry. In the absence of drugs, little cell death was observed (Fig. 7B). Treatment with enzalutamide or DJ160 resulted in an increase in the percentage of cleaved caspase-positive cells in patient samples 3 and 4, whereas no/minimal apoptosis was induced in patients 1 and 2. It therefore appears that compound DJ160 is cytostatic in all patients, even those that appear to be resistant to enzalutamide, and cytotoxic in a proportion of the samples.

## Discussion

Thieno[2,3-*b*]pyridines are promising anticancer therapeutics but little is known about their potential as a therapeutic option for prostate cancer. It has been previously shown that the compounds are effective at inhibiting stem cells derived from the prostate cancer cell line DU145, suggesting that they may be a useful option for the disease (Mastelic *et al.* 2017). The effect of these compounds upon proliferation, motility and cell death was therefore investigated in a range of prostate cell lines representing different stages of the disease. Compounds DJ097, DJ144, DJ145 and DJ160 all contain a 3-substituted arylcarboxamido moiety, which has previously been found to have good activity on other cell lines. DJ154 was chosen to determine the effect of removal of the ketone group by comparison to DJ097. In agreement with previous studies investigating different cancer cell types (e.g. (Leung *et al.* 2014)), we have demonstrated that the thieno[2,3-*b*]pyridines potently inhibit prostate cancer growth.

Interestingly, the prostate cancer lines tested appear to be more sensitive than other cancer types. For example, compound DJ160 was found to have IC_50_ values of >100 nM across various breast cancer cell lines. In contrast, this compound had an IC_50_ of <50 nM in multiple prostate cancer lines (LNCaP, C42 and PC3). Importantly, the thieno[2,3-*b*]pyridines investigated, in particular DJ97 and DJ145, were found to have some selectivity for the prostate cancer cell lines compared to the non-tumorigenic control BPH1. Interestingly, these two compounds are the most chemically similar, differing only in the halogen at the 3-position in the phenylamide ring. In comparison, DJ160 has an additional methyl group at the 2-position in the same ring and this 3-chloro-2-methyl substitution pattern has been shown to induce high levels of cytotoxicity in all cell lines tested (Leung *et al.* 2016, Pilkington *et al.* 2016, van Rensburg *et al.* 2017, Haverkate *et al.* 2021).

Flow cytometry analysis and fluorescence imaging demonstrated that the thieno[2,3-*b*]pyridines promote G2/M arrest and promote multinucleation, similar to studies in breast cancer cell lines (Leung *et al.* 2014, Reynisson *et al.* 2016). As mentioned previously, the thieno[2,3-*b*]pyridines were originally identified in a screen that aimed to identify inhibitors of PLCγ (Reynisson *et al.* 2009). However, thieno[2,3-*b*]pyridines activity does not correlate with PLCγ1/2 expression, nor was PLC activity inhibited by DJ160, suggesting that the cellular effects of the compounds cannot be attributed to the inhibition of PLCγ activity. Additional studies are needed to investigate this further, as it is possible that PLCγ only makes up a small fraction of the overall PLC activity and is therefore being masked by redundancy in the system, but our hypothesis is that the thieno[2,3-*b*]pyridines exert their effects via a different target(s).

Importantly, thieno[2,3-*b*]pyridines have been suggested to bind directly to and regulate the activity of tubulin (Eurtivong *et al.* 2017). It is therefore likely that a number of the described cellular effects induced by the inhibitors are a result of modulation of microtubule polymerisation. For example, the inhibitors were found to promote multinucleation, which can be caused by disruption of microtubule dynamics and this can lead to mitotic catastrophe, activation of caspases and apoptosis (Mc Gee 2015). This is in accordance with the findings presented here, as the compounds were also found to promote caspase-dependent cell death. Furthermore, the thieno[2,3-*b*]pyridines were also found to inhibit cell motility at sub-lethal concentrations of the compounds. Microtubules also play an important role in the intracellular transport of molecules essential for motility and are involved in generating cellular protrusions (Garcin & Straube 2019). The inhibition of cell motility may therefore also be a result of the disruption of microtubule dynamics and experiments to confirm this are warranted. With the exception of DJ160, the compounds’ IC_50_ values do not show a strong correlation with the results of the phenotypic assays; there was poor correlation between IC_50_ value and caspase activity, multinucleation and DNA hypodiploidy (Supplemental Fig. 1). In part, this may be a limitation of specific time points/drug concentrations being chosen for the different experiments but may also reflect polypharmacology.

Compound DJ160 was taken forward for testing in patient-derived explants; pre-clinical physiologically relevant models that recapitulate the tumour microenvironment in a 3D model (Centenera *et al.* 2018). Analysis of caspase 3 cleavage revealed that half of the patient samples exhibited caspase 3 activation in response to both DJ160 and enzalutamide. The absence of caspase 3 activation in some samples might reflect the stage of mitotic catastrophe cells are undergoing rather than their inability to undergo cell death, as only the final stages of mitotic catastrophe are dependent on caspases (Skwarska *et al.* 2007, Vakifahmetoglu *et al.* 2008). Relying on caspase 3 as a marker for assessing the effectiveness of DJ160 as an anticancer drug might therefore under-represent the extent of overall cytotoxic effects. This is important in light of the third type of anticancer impact these compounds exert, which is anti-proliferative. These measurements demonstrated that thieno[2,3-*b*]pyridines potently block cell proliferation, evidenced by consistent decreases in PCNA expression across all explants treated with DJ160, including tumour samples resistant to enzalutamide. Thus, thieno[2,3-*b*]pyridines are effective inhibitors that offer a treatment alternative even in scenarios where tumours have acquired resistance to standard therapies such as antiandrogens.

## Supplementary materials



## Declaration of interest

The authors declare that there is no conflict of interest that could be perceived as prejudicing the impartiality of the work reported.

## Funding

This work did not receive any specific grant from any funding agency in the public, commercial or not-for-profit sector. MA was supported by the Saudi Arabian Cultural Bureau; DL was supported by Prostate Cancer UK (RIA18-ST2-022). GB, RZ and AM were funded by Prostate Cancer UK (RIA15-ST2-014).

## Author contribution statement

MA and DL performed the experiments. MA and GB performed data analysis. MA, DL, AM, RZ, PL, MM, CB, JR and GB designed experiments. MR, LP and DB generated and provided compounds. GB and JR wrote the manuscript. DL, AM, RZ, DB, JR and GB edited the manuscript.

## Data availability

The data that support the findings of this study are available from the corresponding author upon reasonable request.

## Ethics statement

Samples used in this research project were obtained from the Imperial College Healthcare Tissue Bank (ICHTB). ICHTB is supported by the National Institute for Health Research (NIHR) Biomedical Research Centre based at Imperial College Healthcare NHS Trust and Imperial College London. ICHTB is approved by Wales REC3 to release human material for research (22/WA/0214), and the samples for this project (R18041) were issued from sub-collection reference number Uro_MW_13_010.
