# Supplementary material for: Thieno[2,3-b]pyridine compounds potently inhibit prostate cancer growth and motility
Source: Endocr Oncol. 2025 Jul 8;5(1):e240082. doi: 10.1530/EO-24-0082 (PMC12243099; doi:10.1530/EO-24-0082)
Supplement: Supplementary file 1 [file supplementary_materials.pdf]

# *Supplemental Table 1*

**Supplemental Table 1. qPCR primer sequences.**

| qPCR primer | Primer sequence (5’-3’) |
|---|---|
| PCNA_F | GGCCGAAGATAACGCGGATAC |
| PCNA_R | GGCATATACGTGCAAATTCACCA |
| β-actin_F | GGCATCCTCACCCTGAAGTA |
| β-actin_R | GGTCATCTTCTCGCGGTTG |
| L19_F | GCGGAAGGGTACAGCCAAT |
| L19_R | AGCAGCCGGCGCAAA |
| GAPDH_F | ATGGGGAAGGTGAAGGTCG |
| GAPDH_R | GGGGTCATTGATGGCAACAATA |

# *Supplemental Figure 1*

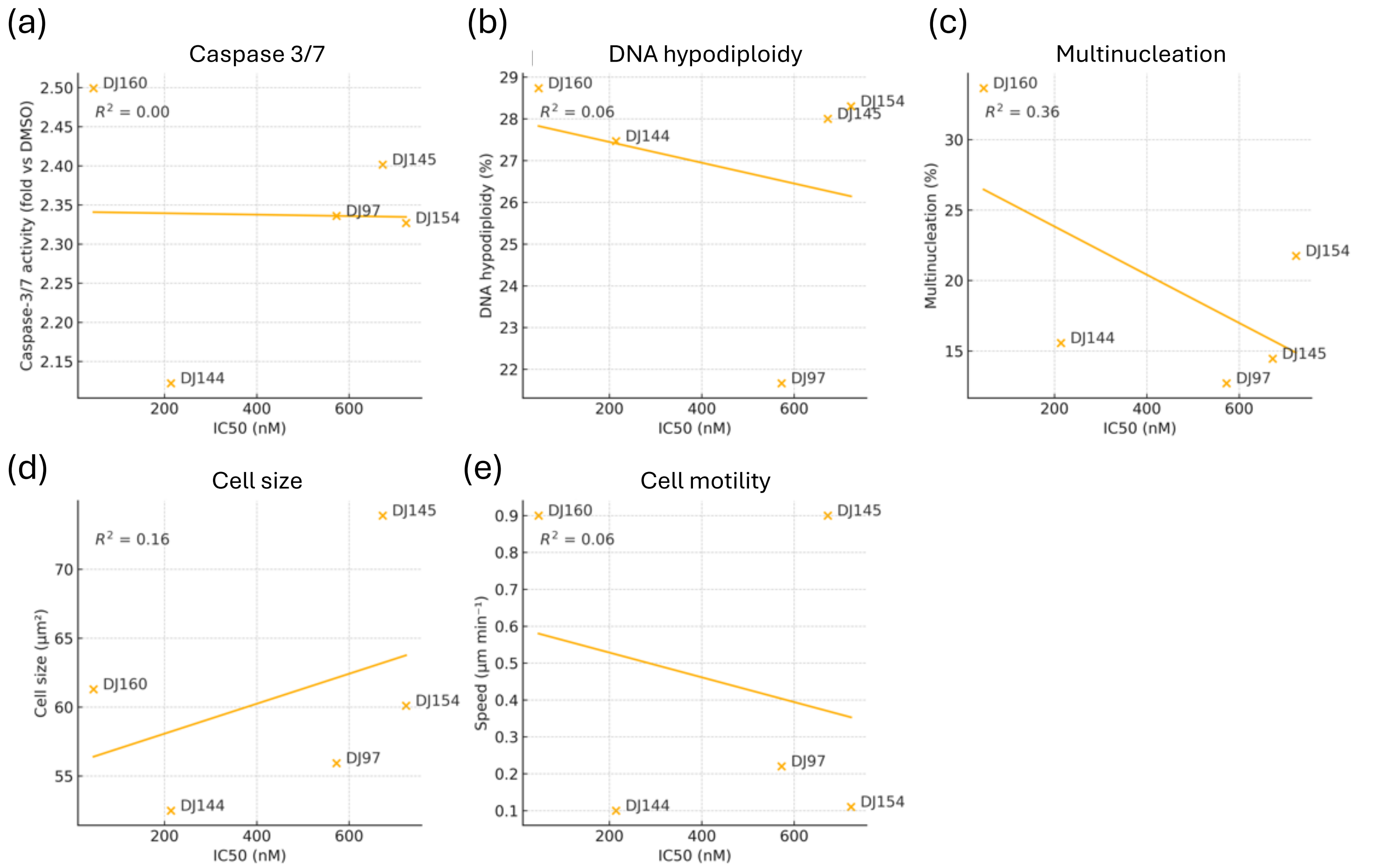


**Supplemental Figure 1. The $IC_{50}$ values for the thieno[2,3-*b*]pyridine compounds show little correlation with the phenotypic assays.** The $IC_{50}$ values were plotted against the results of the (a) caspase 3/7, (b) DNA hypodiploidy, (c) multinucleation, (d) cell size and (e) cell motility data. $R^2$ values are provided to indicate the goodness-of-fit between variables
